# WISC-IV performance of children with Chronic Tic Disorder, Obsessive–Compulsive Disorder and Attention-Deficit/Hyperactivity Disorder: results from a German clinical study

**DOI:** 10.1186/s13034-021-00392-4

**Published:** 2021-08-31

**Authors:** Sina Wanderer, Veit Roessner, Anja Strobel, Julia Martini

**Affiliations:** 1 Department of Child and Adolescent Psychiatry, Faculty of Medicine, Carl Gustav Carus University Hospital, Technische Universität Dresden, Fetscherstraße 74, 01307 Dresden, Germany; 2 Department of Psychology, Chemnitz University of Technology, Wilhelm-Raabe-Str. 43, 09120 Chemnitz, Germany; 3 Department of Psychiatry & Psychotherapy, Faculty of Medicine, Carl Gustav Carus University Hospital, Technische Universität Dresden, Fetscherstr. 74, 01307 Dresden, Germany

**Keywords:** Wechsler Intelligence Scale for Children for Children (WISC), Chronic Tic Disorder (CTD), Obsessive–Compulsive Disorder (OCD), Attention-Deficit/Hyperactivity Disorder (ADHD)

## Abstract

**Background:**

Chronic Tic Disorder (CTD), Obsessive–Compulsive Disorder (OCD) and Attention-Deficit/Hyperactivity Disorder (ADHD) are complex neuropsychiatric disorders that frequently co-occur. The aim of this study was to examine WISC-IV performance of a clinical cohort of children with CTD, OCD and/or ADHD.

**Methods:**

N = 185 children aged 6 to 17 years from Germany with CTD, OCD and/or ADHD were examined with the WISC-IV that comprises four index scores (VCI: Verbal Comprehension Index, PRI: Perceptual Reasoning Index, WMI: Working Memory Index, PSI: Processing Speed Index) and a Full Scale Intelligence Quotient (FSIQ). WISC-IV profiles of children with CTD-only, OCD-only, ADHD-only, CTD+ADHD, CTD+OCD and CTD+OCD+ADHD were compared with the WISC-IV norm (N = 1650, M = 100 and SD = 15) and among each other.

**Results:**

Unpaired t-tests revealed that children with ADHD-only showed significant lower PSI scores, whereas children with CTD-only and OCD-only had significant higher VCI scores as compared to the German WISC-IV norm. One-way ANOVA revealed that children with ADHD-only showed significant lower WMI scores as compared to children with CTD+OCD.

**Conclusions:**

We were able to confirm previous evidence on WISC-IV profiles in ADHD in a German clinical sample and contribute new findings on cognitive performance in children with (non-)comorbid CTD and OCD that have to be seen in light of the study’s limitations.

## Background

Chronic Tic Disorder (CTD) (incl. Tourette Syndrome, TS), Obsessive–Compulsive Disorder (OCD) and Attention-Deficit/Hyperactivity Disorder (ADHD) are common neurodevelopmental disorders in childhood which tend to occur frequently as comorbid conditions [1–4].

Tics are sudden, involuntary movements or vocalizations that wax and wane in severity, frequency and complexity [5]. The mean onset of CTD is at the age of 6 years. In about 0.5% to 3% of all children tics become chronic and last longer than 1 year. Boys are about three to four times more often affected than girls [6, 7]. The most common comorbidity of CTD is ADHD (up to 60%), followed by OCD (about 20 to 30%) [8, 9].

OCD is characterized by recurrent, distressing thoughts and/or compulsive acts [5] with a prevalence of 1 to 3% in childhood and adolescence [10]. Compared to the onset of CTD the average onset of OCD is later in childhood and the gender ratio is nearly balanced [3, 11]. In children with OCD about 12% fulfil the diagnostic criteria of CTD and approximately 8% suffer also from ADHD [3, 12]. ADHD is characterized by the core symptoms inattention, hyperactivity and impulsivity and at least some of these symptoms have to be present before the age of 7 years for a period of at least 6 months [5]. About 5–7% of children suffer from ADHD [13, 14] with considerably more boys than girls being affected (sex ratio: 3–4:1) [15]. Children with a primary diagnosis of ADHD often fulfill also the diagnostic criteria of CTD (about 20%) or OCD (notable variability in ADHD-OCD co-occurrence in pediatric samples) [2, 3].

The high rate of comorbidity suggests a shared neurobiological basis [4, 16, 17] and may be associated with stronger impairment and more performance difficulties [18–22]. Underlying neurobiological mechanisms have been investigated in recent decades, though much remains to be uncovered (e.g. [3]). Cognitive and neuropsychological deficiencies include inhibited executive functioning (i.e. planning, working memory, impulse control), verbal and non-verbal memory impairment, and other intellectual deficiencies. These accompanying symptoms might affect school performance, learning, as well as social and emotional abilities. For example, ADHD is thought to result mainly from prefrontal-striatal dysfunction, leading to impairments in executive functioning [23], which in turn leads to poor performance in processing speed, inhibition, working memory, verbal fluency, and shifting [24]. The relationship between etiological models of CTD and OCD and intelligence or rather the role of intelligence deficits in CTD and OCD is rarely investigated (e.g. [25]). Thus it is not clear whether they may be seen as a part of a common etiology, as a consequence or both.

The examination of strengths and difficulties in intellectual ability in children with CTD, OCD and/or ADHD is crucial, because this is important for academic development, social functioning and well-being [26–28]. Different models and intelligence tests are available for the assessment of intellectual ability (e.g. Ravens Progressive Matrices, Wilde Intelligence Test) with the Wechsler Intelligence Scale for Children (WISC, according to the Carrol-Horn-Cattell-Model) being one of the most frequently used instruments in both clinical practice and research [29–31].

Previous studies on intelligence profiles in children with CTD and/or OCD are rare and studies on ADHD are characterized by large methodological differences with regard to the use of different WISC versions (e.g. WISC-R, WISC-III, WISC-IV) [29–31], different study designs and the examination of different diagnostic and control groups.

As illustrated in Table 1, previous studies investigated intelligence profiles with or without consideration of comorbid disorders or explicitly focused on comorbidity [4, 32–39]. Overall, there is ample evidence that children with ADHD show impairments in intellectual ability as compared to control groups (for meta-analysis see [40]). Regards of the WISC edition [29–31] the most studies showed significantly lower values in the WISC scores currently called Processing Speed Index (PSI) and Working Memory Index (WMI) in children with ADHD [32, 34, 36, 38, 40, 41] and it is even discussed to consider the WISC profiles in the clinical diagnostic process of ADHD [38, 40, 42].

**Table 1:** Selected studies on WISC profiles in children with CTD (including TS) OCD and/or ADHD

	Study	Instrument	Age (in years)	Country	Selected results focusing WISC scores/ profiles in ADHD, OCC and CTD
Frazier et al. [40]	Meta-analysis on 123 studies	WISC-III, WISC-R	Different	Different	Effect sizes for FSIQ were significantly different between ADHD and healthy subjects (weighted d = .61); VIQ (d = .67) and PIQ (d = .58) were significantly sensitive to ADHD
Moura et al. [68]	Children with (n = 98) and without (n = 81) ADHD matched by age and gender	WISC-III	6–12	Portugal	Significant index scores discrepancies between subjects with ADHD and control group in FSIQ, VIQ, PIQ; FDI showed the highest diagnostic accuracy to discriminate children with ADHD
Theiling et al. [66]	Adults with (n = 116) and without (n = 116) ADHD, randomly matched controls	WAIS-IV	16–71	Germany	Adults with ADHD show significant decrements in subtests WMI and PSI, and a higher GAI in comparison with the FSIQ; deficits can also be found in adults with ADHD and WAIS-IV reliability differentiates between patients and controls
Schmidtendorf et al. [35]	Children and adolescents (n = 433) with AD(H)D, children and adolescents with anxiety or other emotional disorder (N = 41)	WISC-IV	6–16.5	Germany	Significant deficit in PSI in total sample and also in subsample cleared for comorbidities (N = 117), WMI deficits seem to occur only if comorbid disorders are present Similar profiles of the AD(H)D-only and clinical control group
Beers et al. [46]	Unmedicated subjects with OCD (n = 21), matched controls (n = 21)	WISC-III	OCD: 12.3 (2.9) controls:12.2 (2.9)	USA	No cognitive deficits in Digit Span and Block Design in subjects with OCD
Hagberg et al. [34]	Subjects with ASD a/o ADHD (n = 40), n = 21 subjects from the community	WISC-III	6.4–9.9	Sweden	FSIQ was lower than population norms and similar across diagnostic groups (ASD, ADHD) and settings (clinic, community)
Walg et al. [38]	Male subjects with AD(H)D (n = 50), male subjects with other mental disorder (n = 54)	WISC-IV	7–16	Germany	Significant lower PSI in subjects with AD(H)D vs. controls
Calhoun and Mayers [32]	Subjects with ADHD (n = 431), ADD (n = 134) and other diagnoses	WISC-III	6–16	USA	PSI and FDI scores in subjects with AD(H)D below the group mean and lower than VCI and POI
Khalifa et al. [20]	Children with TS from the general population (n = 25)	WISC-III	7–15	Sweden	High variation in WISC-III profiles of subjects with TS (VCI,FDI,PSI were 2.5–5 points below the average)
Mayers and Calhoun [47]	Comparison of n = 586 WISC-III profiles and n = 118 WISC-IV profiles of subjects with ADHD and normal intelligence	WISC-III, WISC-IV	6–16	USA	Significant lower FDI,WMI,PSI than VCI and POI/PRI in subjects with ADHD; similar profiles of WISC-III and WISC-IV
Rizzo et al. [4]	Subjects with TS only (n = 20), ADHD only (n = 20), TS+ADHD (n = 20, controls (n = 20)	WISC-R	6–16	Italy	No significant differences in FSIQ,VIQ,PIQ between TS-only and controls Significant lower FSIQ,VIQ,PIQ in subjects with ADHD-only and TS+ADHD as compared to controls
Debes et al. [33]	Subjects with TS and comorbid ADHD and/or OCD (n = 266), healthy matched controls n = 80	WISC-III	10–16	Denmark	Lower IQ scores in subjects with TS compared to controls and the general population, except for children with TS+OCD who scored higher in FSIQ
de Groot et al. [45]	Children with TS (92) grouped by the presence and absence of OCD and/or AHDH: TS (n = 34), TS+OCD (n = 14), TS+ADHD (n = 23), TS+OCD+ADHD (n = 21)	WISC-R WAIS-R	6–18	Canada	Significant group effect for FSIQ TS > TS+OCD VIQ and PIQ significant multivariate effect and significant univariate effect for VIQ (TS > TS+OCD, TS+OCD+ADHD)
Schuerholz et al. [43]	TS only (n = 21), TS + ADHD (n = 19), TS ± ADHS (ADHS status not strongly confirmed, n = 25), unaffected siblings (n = 27)	WISC-R	6–14	North America	Higher FSIQ in TS-only as compared to TS+ADHD and unaffected siblings (wide variance of FSIQ scores)
Shin et al. [36]	Subjects with OCD (n = 17), TD (n = 21), ADHD (n = 25), Depression (n = 20), healthy controls (n = 23)	WISC-R	6–16	Korea	Significant lower FSIQ,VIQ,PIQ in clinical groups vs. controls (all groups within the average range) Subjects with OCD tended to have higher VIQ than subjects with TD, ADHD and depression, lower PIQ in OCD than in TD and depression
Termine et al. [37]	Unmedicated male subjects with TS (n = 13), TS+ADHD (n = 8), ADHD (n = 39), controls healthy (n = 66)	WISC-III	6–15	Italy	Significant lower scores in components of the PIQ and VIQ (Block Design, Vocabulary) in TS a/o ADHD as compared to healthy controls
Yeates and Bornstein [39]	Subjects with TS (n = 46), TS+ADHD (n = 36)	WISC-R	6–18	USA	Subjects with TS+ADHD did not differ from subjects with TS-only in FSIQ,VIQ,PIQ

CTD: Chronic Tic Disorder; TD: Tic Disorder; TS: Tourette syndrome; OCD: Obsessive–Compulsive Disorder; AD(H)D: Attention-Deficit (Hyperactivity) Disorder; WSIC-III: Wechsler Intelligence Scale for Children, 3rd edition; WISC-IV: Wechsler Intelligence Scale for Children, 4th edition; WISC-R: Wechsler Intelligence Scale for Children–Revised; FSIQ: Full Scale Intelligence Quotient; VIQ: Verbal Intelligence Quotient; PIQ: Performance Intelligence Quotient; VCI: Verbal Comprehension Index; PRI: Perceptual Reasoning Index; WMI: Working Memory Index; PSI: Processing Speed Index; FDI: Freedom From Distractibility; POI: Perceptual Organization Index; PRI: Perceptual Reasoning Index

With regard to CTD there is a considerable lower number of studies that revealed heterogeneous results and high variations in WISC profiles [20, 33]. Several studies have identified impaired intellectual functioning unique to the comorbidity of CTD TS and ADHD/OCD (e.g. [4, 37, 43–45]). For example, Debes et al. (2011) investigated the intellectual ability with the WISC-III in a clinical sample of N = 266 children with TS and comorbid ADHD and/or OCD. They found WISC-III index scores to be below average in children with TS with and without comorbidities. The only exception were children with TS+OCD who scored higher in Full Scale IQ (FSIQ) as compared to the children of the other clinical groups (TS-only, TS+ADHD, TS+ADHD+OCD) [33]. Another study by de Groot et al. found significantly lower Verbal IQ and Performance IQ scores in children with TS+OCD and TS+OCD+ADHD and lower FSIQ scores in children with TS+OCD as compared to TS (without OCD and ADHD) [45]. Overall, studies on cognitive performance in children with CTD with and without comorbid disorders revealed heterogeneous results and a large variation in WISC scores [4, 20, 33, 36, 39].

Finally, only a few studies investigated WISC profiles in children with OCD. For instance, in a study by Beers et al. children with OCD did not show cognitive impairment in WISC-III scores [46] whereas Shin et al. reported performance deficits in the WISC-R subtests assessing perceptual organization ability [36].

To summarize, there is ample evidence on WISC performance of children with ADHD, but WISC profiles in children with CTD and OCD were rarely examined. Comparison of studies is hampered by different study designs, substantial methodological differences (e.g. different WISC versions, age groups, sampling procedures) and different consideration of comorbidity (see Table 1). The present study aims to investigate intellectual ability in children with CTD, OCD and/or ADHD, that commonly co-occur (especially) in clinical settings [1–3]. Here, the examination of intelligence profiles in children with CTD and/or OCD is of particular interest because of the low number of studies [4, 20, 33, 36, 39, 46] that revealed heterogeneous results and a large variation in WISC scores.

The Department of Child and Adolescent Psychiatry (CAP) Dresden offers special consultation hours for these disorders and we were therefore able to analyze clinical data of a substantial number of affected children. Due to our comprehensive and standardized assessment process, we were furthermore able to identify comorbid and non-comorbid disorders. As we used field data, no healthy control group was assessed. Using the clinical data from the CAP Dresden electronic health records, this study was conducted in order to evaluate whether or not children with only one (namely CTD-only, OCD-only or ADHD-only) differ from those with comorbid disorders (e.g. CTD+ADHD, CTD+OCD+ADHD) in intellectual functioning. For this, WISC-IV profiles of different groups will be compared to the German WISC-IV norm and among each other. The study was also undertaken as previous studies on intelligence profiles in CTD and OCD produced conflicting results and few have been published from Europe. Based on a large body of literature, we hypothesized that ADHD is associated with deficits in WISC-IV WMI and PSI scores [35, 38, 41, 47]. Due to the heterogeneous studies on intelligence profiles in children with CTD and/or OCD, no specific hypotheses were formulated.

## Methods

The Department of Child and Adolescent Psychiatry (CAP) of the University Hospital Carl Gustav Carus at the Technische Universität Dresden (Germany) offers special consultation hours for children with CTD, OCD and ADHD. Families mainly come from the area of Dresden, but also from all over Germany. All children pass an extensive diagnostic procedure including a physical examination, a comprehensive anamnesis, several clinical assessments (Child Behavior Checklist [48], Strengths and Difficulties Questionnaire [49]; and for specific diagnoses, e.g. Yale Global Tic Severity Scale [50]; Yale Brown Obsessive Compulsive Scale (CY-BOCS) [51, 52], Conners’ Scales [53, 54]), and if necessary, neuropsychological tasks (incl. WISC-IV) and behavioral observation at home and in school. The diagnostic procedure at CAP can comprise several appointments (usually 5–7 appointments). All children receive an ICD-10 diagnosis [5] based on the assessments and the consensus of a multi-professional team (incl. physicians, psychologists, social worker).

### Participants

The study was based on data from an accumulated clinical sample of children who were seen at special consultation hours for CTD, OCD and ADHD at the CAP Dresden in the period between 01.01.2010 and 31.03.2016. As these are field data, no healthy control group was assessed.

For this study, the following inclusion and exclusion criteria were defined:

Inclusion criteria:

- children aged 6;0 to 16;11 years
- diagnosis of CTD (ICD-10: F95.1 and F95.2), OCD (ICD-10: F42.-) and/or ADHD (ICD-10: F90.- and F98.8; random sample, see below) [5]
- WISC-IV information drawn from the CAP Dresden electronic health records.

Exclusion criteria:

- psychiatric or developmental disorders other than CTD, OCD and ADHD, (e.g. adjustment disorder, anxiety disorder, mood disorder, autism spectrum disorder)
- no WISC-IV information in the CAP Dresden electronic health records.

### Sample

**Fig. 1:**
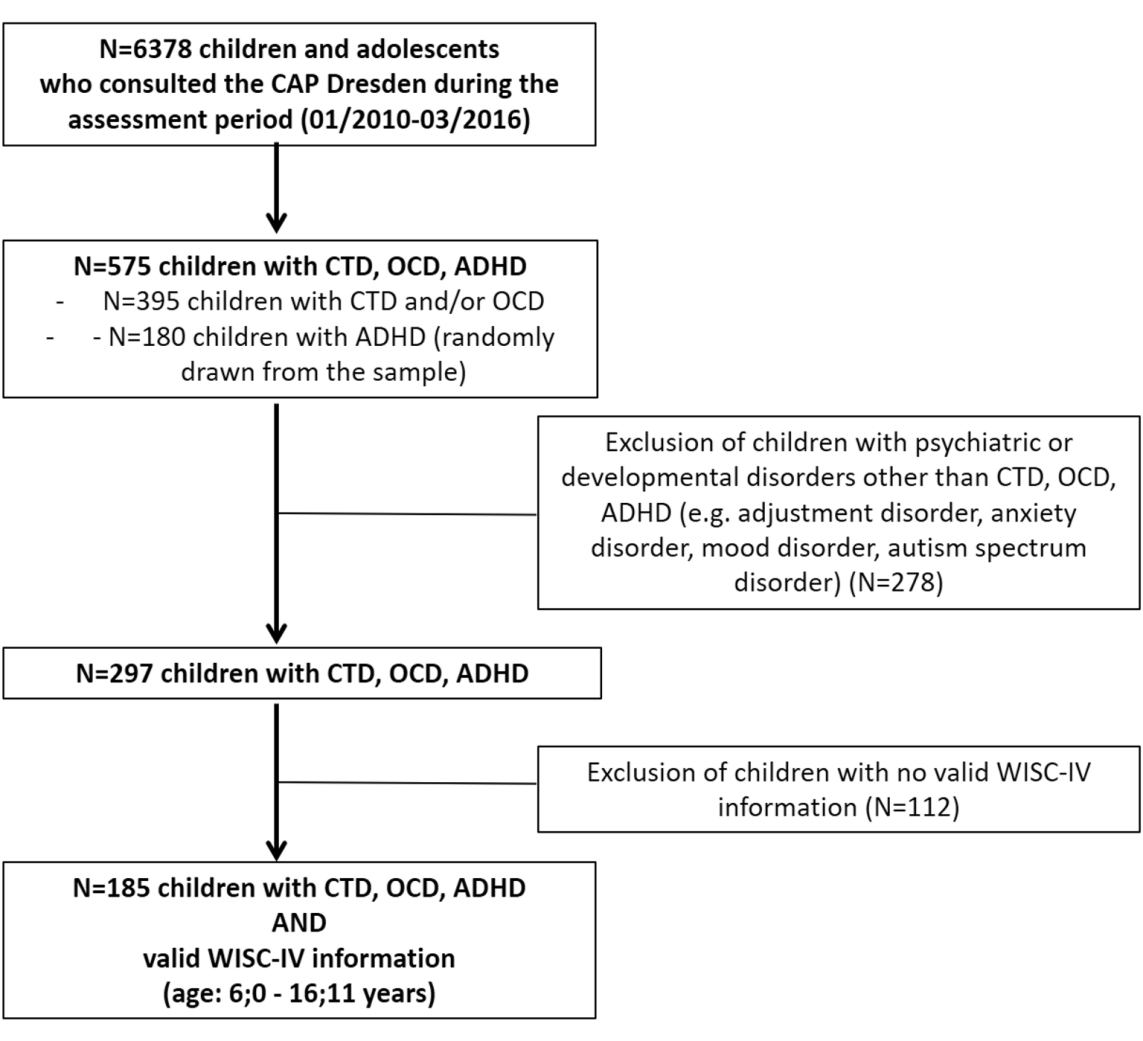
Chart of the sample selection. CTD: Chronic Tic Disorder; OCD: Obsessive–Compulsive Disorder; ADHD: Attention-Deficit/Hyperactivity Disorder; WISC-IV: Wechsler Intelligence Scale for Children, 4th edition

Overall, N = 6378 children and adolescents with various problems and diagnoses consulted the CAP Dresden during the assessment period (see Fig. 1).

Our foremost target was the investigation of WISC-IV profiles in children with (comorbid) CTD and/or OCD. Thus, all children with a diagnosis CTD and/or OCD (N = 395) (with and without other diagnoses) were selected from the CAP Dresden electronic health records in the first step.

Given the conclusive evidence on intelligence profiles in ADHD (numerous comprehensive studies and reviews available, already) and the large number of children with ADHD who consulted the CAP Dresden (N = 689) during the assessment period, we decided to randomly draw a subsample of N = 180 children with ADHD to ensure comparable group sizes.

Thus, N = 575 children with CTD, OCD and/or ADHD were available for further consideration.

In a next step, we excluded other comorbidities (e.g. adjustment disorder, anxiety disorder, mood disorder, autism spectrum disorder) from this sample to disentangle the specifics of intellectual ability in CTD, OCD and ADHD, resulting in a sample of N = 297 children.

Finally, only cases with information on WISC-IV and within the approved age range of the WISC-IV (6;0 to 16;11 years) were included in our analyses resulting in N = 185 children as final clinical sample.

### Ethics approval and consent to participate

Due to anonymous data selection from the health records informed consent from the participants was not required. The study was approved by the institutional review board of the Medical Faculty of the Technische Universität Dresden (No: EK31012016) and has been performed in accordance with the World Medical Association Declaration of Helsinki [55].

### Measurement

The Wechsler Intelligence Scale for Children Fourth Edition (WISC-IV) [31] measures intellectual ability. It comprises ten core subtests and four index scores standardized for sex and age: Verbal Comprehension Index (VCI) measuring the ability of verbal reasoning and acquired knowledge, Perceptual Reasoning Index (PRI) assessing perceptual organization and logical reasoning, Working Memory Index (WMI) measuring attention and working memory, and Processing Speed Index (PSI) assessing speed of mental and fine motor processing. Furthermore, a Full Scale Intelligence Quotient (FSIQ) can be analyzed.

Analysis was based on the German normative sample of N = 1650 children and adolescents from German speaking areas (Germany, Swiss, Austria) aged 6;0 to 16;11 years [56]. Data from the norm sample were collected between 2005 and 2006 and has been stratified by gender, age, school type, therefore being commensurate with the German census. On the basis of this norm sample, age-specific WISC-IV index scores and FSIQ were calculated and then standardized on M = 100 and SD = 15. As the authors did not find any gender-specific differences in FSIQ, the index scores and FSIQ were calculated on the basis of age-specific norm tables.

The internal reliability coefficients (measured with the split-half and retest method) for the index scores and FSIQ range from r = 0.87 (Processing Speed) to r = 0.97 (FSIQ) [56].

Unfortunately, there was no systematic information available in the CAP Dresden electronic health records regarding medication use at WISC-IV assessment. Although we assume that most of the children receive medication only after the entire diagnostic procedure at CAP was finished, it cannot be ruled out that a small number of children cannot be ruled out that a small number of childrean was already under medication at WISC-IV assessment.

### Statistical analysis

SPSS 23.0 [57] and STATA 14 [58] were used for analyses. The information about diagnoses, age, gender, WISC-IV index and FSIQ score of the selected participants was read out from the CAP Dresden electronic health records and entered into SPSS 23.0 [57] anonymously and manually.

Due to their psychiatric diagnoses, children were assigned to one of the following groups: CTD-only (n = 46), OCD-only (n = 21), ADHD-only (n = 45), CTD+ADHD (n = 35), CTD+OCD (n = 20), OCD + ADHD (n = 4), CTD+OCD+ADHD (n = 14). Since, there were only four children with OCD+ADHD this subgroup was insufficiently sized/powered and therefore excluded from further analyses.

Pearson Chi^2^ tests and one-way ANOVA were performed to test whether sample characteristics (age and gender) differed between the groups. Independent t-tests were calculated to test differences between the groups and the German WISC-IV norm. For the calculation N, M, SD of the observed data from our sample were compared with N, M, SD that were derived from the WISC-Manual (e.g. N = 1650, M = 100, SD = 15) using the following formula $t = \frac{Mnorm - Mobservation}{{\sqrt {\frac{{SDnorm^{2} }}{Nnorm} + \frac{{SDobservation^{2} }}{Nobservation}} }}$.

A one-way ANOVA was conducted to compare the groups CTD-only, OCD-only, ADHD-only, CTD+ADHD, CTD+OCD and CTD+OCD+ADHD with each other. A significance level of alpha = 0.05 was adopted with Bonferroni test as post hoc test.

## Results

### Sample description

Table 2 presents descriptive data on age and gender distributions in the respective groups (CTD-only, OCD-only, ADHD-only, CTD+ADHD, CTD+OCD and CTD+OCD+ADHD) and revealed a significant difference in mean age (F = 5.662, p < 0.001).

**Table 2:** Sample description (mean age, sex distribution and WISC-IV mean scores) of children with pure and comorbid CTD, OCD and ADHD

	CTD-only (n = 46)		OCD-only (n = 21)		ADHD-only (n = 45)		CTD+ADHD (n = 35)		CTD+OCD (n = 20)		CTD+OCD+ADHD (n = 14)				
	N	%	N	%	N	%	N	%	N	%	N	%	Chi^2^	p	
**Gender** Female	9	19.6	7	33.3	6	13.3	5	14.3	5	25	3	21.4	4.702	0.453	
Male	37	80.4	14	66.7	39	86.7	30	85.7	15	75	11	78.6			
Female:male	1:4.1		1:2		1:6.5		1:6		1:3		1:3.7				

	**M (SD)**		**M (SD)**		**M (SD)**		**M (SD)**		**M (SD)**		**M (SD)**		**ANOVA**	**P**	**Bonferroni**
Age	10.9 (2.7)		11.6 (2.6)		9.3 (2.2)		9.1 (2.6)		11.7 (2.8)		10.4 (2.3)		**F = 5.662; df1 = 5, df2 = 175**	**< 0.001**	**ADHD-only, CTD+ADHD < CTD-only, OCD-only, CTD+OCD**

**WISC-IV**	**M (SD)**		**M (SD)**		**M (SD)**		**M (SD)**		**M (SD)**		**M (SD)**		**ANOVA**	**P**	**Bonferroni**
VCI	107 (14.3)		108 (12.5)		103 (13.1)		104 (14.9)		104 (15.4)		102 (12.9)		F = 0.710; df1 = 5, df2 = 175	0.616	
PRI	105 (15.2)		106 (13.8)		102 (14.8)		105 (14.9)		105 (15.5)		105 (17.8)		F = 0.336; df1 = 5, df2 = 175	0.891	
WMI	102 (11.7)		100 (13.1)		96 (11.6)		102 (14.0)		106 (12.2)		100 (12.3)		**F = 2.335; df1 = 5, df2 = 175**	**0.044**	**ADHD-only < CTD+OCD**
PSI	98 (14.2)		99 (15.0)		94 (10.9)		96 (12.9)		104 (12.5)		99 (14.7)		F = 1.653; df1 = 5, df2 = 174	0.148	
FSIQ	105 (13.8)		105 (11.1)		99 (11.9)		102 (13.8)		106 (14.2)		102 (13.2)		F = 1.309; df1 = 5, df2 = 174	0.262	

CTD: Chronic Tic Disorder; OCD: Obsessive–Compulsive Disorder; ADHD: Attention-Deficit/Hyperactivity Disorder; M: Mean; SD: Standard Deviation; VCI: Verbal Comprehension Index; PRI: Perceptual Reasoning Index; WMI: Working Memory Index; PSI: Processing Speed Index; FSIQ: Full Scale Intelligence Quotient

Post hoc Bonferroni tests showed that children with ADHD-only and CTD+ADHD were significantly younger than children with CTD-only, OCD-only and CTD+OCD. There was no significant difference in sex distribution between the groups.

### Comparison of WISC-IV profiles in children with CTD, OCD and/or ADHD with the WISC-IV norm

**Fig. 2:**
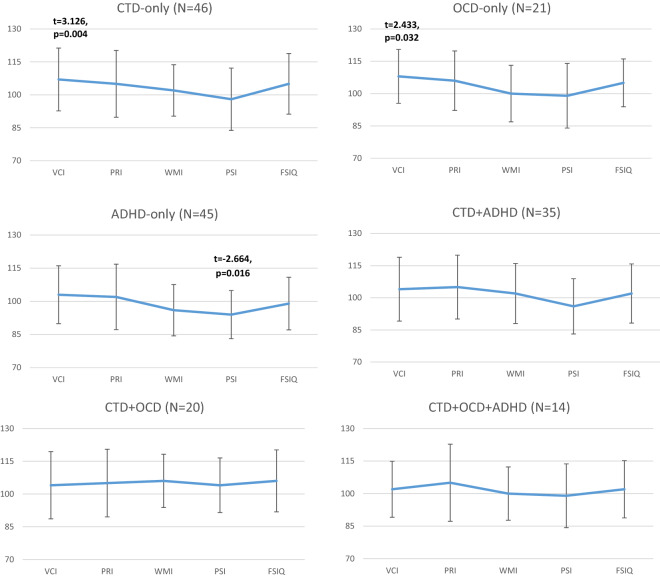
WISC-IV profiles in children with Chronic Tic Disorder (CTD), Obsessive–Compulsive Disorder (OCD) and/or Attention-Deficit/Hyperactivity Disorder (ADHD) compared to the WISC-IV norm sample (N = 1650, M = 100, SD = 15) (significant results from paired t-tests with Bonferroni correction). VCI: Verbal Comprehension Index; PRI: Perceptual Reasoning Index; WMI: Working Memory Index; PSI: Processing Speed Index; FSIQ: Full Scale Intelligence Quotient

Figure 2 shows the mean WISC-IV profiles of children with CTD-only, OCD-only, ADHD-only, CTD+ADHD, CTD+OCD and CTD+OCD+ADHD as compared to the WISC-IV norm (N = 1650, M = 100, SD = 15). As expected, children with ADHD-only scored significant lower in PSI (t = − 2.664, df = 1693, p = 0.016) as compared to the WISC-IV norm. For children with CTD-only (78% where diagnosed with TS) and OCD-only significant higher mean scores in VCI (CTD-only: t =   3.126, df = 1694, p < 0.004, OCD-only t = 2.433, df = 1669, p = 0.032) were found when comparing profiles with the WISC-IV norm. Interestingly, children with comorbid disorders (CTD+ADHD, CTD+OCD, CTD+OCD+ADHD) showed no significant deviations from the WISC-IV norm.

### Comparison of WISC-IV profiles in children with CTD, OCD and/or ADHD

ANOVA revealed a significant group difference in WMI (F = 2.335, df1 = 5, df2 = 175, p = 0.044) and post hoc Bonferroni test showed that children with ADHD-only had a significantly lower mean WMI score than children with CTD+OCD. No significant differences between the groups with regard to the WISC-IV index scores VCI, PRI and PSI as well as FSIQ were found.

## Discussion

This study investigated WISC-IV profiles in a German clinical sample of children with comorbid and non-comorbid CTD, OCD and/or ADHD who consulted the CAP Dresden (Germany) during the study period from 01.01.2010 until 31.03.2016. In accordance with the earlier onset-age (1) children with ADHD-only and CTD+ADHD were significantly younger than children with CTD-only, OCD-only and CTD+OCD [6, 11]. However, no age or gender related bias was assumed for this study, since the WISC-IV index and FSIQ scores were standardized. (2) When comparing WISC-IV profiles of the considered groups to the German WISC-IV norm, unpaired t-tests revealed that children with ADHD-only showed significant lower PSI scores, whereas children with CTD-only and OCD-only had significant higher VCI scores. (3) ANOVA revealed that children with CTD+OCD scored significantly higher in WMI than children with ADHD-only.

For the discussion of our results, it is important to consider that previous studies followed different methodological approaches (e.g. different WISC-versions, age groups, sampling procedures, consideration of comorbidities).

The result that children with ADHD-only showed significantly lower performances in PSI as compared to the WISC-IV norm (t-tests) and as compared to children with CTD+OCD (ANOVA) is in accordance with previous studies [35, 38, 41, 47] and can be interpreted in a similar direction. The results were in line with another German study of Schmidtendorf et al. who reported significant deficits in PSI and WMI in children with ADHD (N = 433) and furthermore significant deficits in PSI in the subsample of ADHD cleared of comorbidities [35].

As already mentioned, some clinicians even discuss to consider the WISC-IV profiles in the clinical diagnostic process of ADHD [38, 40, 42, 59]. However, it is important to consider that these empirical findings were found at a group level. WISC profiles should be used for individual diagnostic.

When comparing WISC-IV profiles of the considered groups to the German WISC-IV norm, unpaired t-tests revealed that children with CTD-only had significant higher VCI scores. As other studies did not find above-average VCI scores in children with CTD, we have to discuss this result with caution. Other studies that examined cognitive performance in children with CTD (with and without comorbid disorders) revealed heterogeneous results and a large variation in WISC scores [4, 20, 33, 36, 39]. Rizzo et al. found no significant differences in FSIQ between children with TS-only and controls and significant lower FSIQ in subjects with TS+ADHD [4] whereas Debes et al. found lower IQ scores in subjects with TS-only compared to controls and the general population, except for children with TS+OCD who scored higher in FSIQ [33]. Overall, we cannot rule out that our result might be partially due to a selection bias (e.g. fewer WISC scores of children with severe and impairing CTD available from our CAP electronic health records). However, selection bias would likely also pertain WISC performance on the other WISC scores. Moreover, large standard deviations indicate that at least a meaningful proportion of children with CTD performed well during the WISC-IV examination. Further studies are needed to examine intelligence profiles in children with CTD, including TS.

As already mentioned, only a few previous studies investigated WISC-profiles in children with OCD [36, 46]. The present study revealed that children with OCD presented higher VCI scores as compared to the WISC-IV norm. Although an overestimation of the mean WISC scores due to selection bias (see below) cannot be ruled out verbal intelligence performance seems to be rather good within the overall intelligence profiles of children with OCD. Our results were in line with the study by Shin et al. who examined subjects with OCD, TD, ADHD, depression and healthy controls and found that subjects with OCD tended to have higher verbal ability (Verbal Intelligence Quotient) as compared to children of the other clinical groups and similar verbal IQ as compared to the healthy control group [36]. Also, children in a study of Beers et al. performed as good as healthy children in WISC-III and other neuropsychological tests. The authors investigated a pediatric sample of OCD children (age: M = 12.03, SD = 2.9) and concluded that although OCD is associated with central nervous dysfunction, this does not interfere with cognitive ability at an early stage of illness [46]. Given the association between intelligence performance and the development of OCD symptoms [60, 61], more research is needed to investigate the prospective relations between intelligence profiles and OCD.

Interestingly, children with comorbid disorders (CTD+ADHD, CTD+OCD, CTD+OCD+ADHD) showed no significant deviations from the WISC-IV norm in our sample. This result contradicts the common assumption that children suffering from comorbid disorders may have more severe impairments (e.g. cognitive abilities, social functioning [18, 21, 22]), but must be discussed in light with the limitations of our study (see below).

Interestingly, the result that children with CTD+OCD showed significantly higher scores in WMI as compared to ADHD-only (ANOVA: F = 2.335, p = 0.044) was in line with the study of Debes et al. who reported that children with TS+OCD scored higher in FSIQ than the other groups (TS, TS+ADHD and TS+ADHD+OCD) [33]. Regarding ADHD the literature is inconsistent pertaining the relation of comorbidity and cognitive impairments and our study did not show lower WISC performance in children with comorbid ADHD plus OCD and/ or CTD. This is in line with the results of Moura et al. who did not find additional neurocognitive impairments in children with ADHD and comorbid developmental dyslexia as compared to children with ADHD-only [24]. Shanahan et al. argued that processing speed might be a shared cognitive risk factor for both disorders that may help explain the comorbidity of reading disability and ADHD and examined a range of speeded tasks in reading disorder, ADHD and reading disorder+ADHD. They found that the comorbid group did not differ significantly in processing speed from the other groups [62].

On the other hand, Roessner et al. [8] investigated different domains of executive functions (using different tasks e.g. Wisconsin Card Sorting Test, Matching Familiar Figures Test and Stroop Test) in children with non-comorbid and comorbid ADHD and CTD and found that ADHD was associated with neuropsychological performance deficits especially in case of comorbid CTD+ADHD. Thus, further research is necessary to investigate how comorbid conditions are related to cognitive dysfunctions and in particular, working strategies that may be used by the affected children to deal or rather compensate their difficulties [8].

### Strengths and limitations

This study investigated WISC-IV profiles in children with CTD, OCD and/or ADHD considering their co-occurrence. It was based on data from an accumulated German clinical sample of children who were seen at special consultation hours of the CAP Dresden for CTD, OCD and ADHD. So it is one of the first clinical studies in Europe focusing on intellectual profiles of these three disorders (pure and in co-occurance) and controlling for further comorbidities.

As these are field data, no healthy control group was examined since only those children who are under suspicion for a psychiatric diagnosis are further examined at our CAP with the WISC-IV. For this reason, we used the norm data for comparison and we also compared our results with other studies from different countries and various study designs (Table 1). Assuming a theoretical M = 100 and SD = 15 for the control group may produce some bias in the statistical analysis (e.g. many SDs were below the theoretical SD = 15, see Table 2). Further studies should include a control group to allow more robust statistical analyses on cognitive functioning in typically developing children vs. children with CTD, OCD and/ or ADHD (e.g., logistic regression analysis, ROC curve analysis).

Unfortunately, there was no systematic information available regarding medication use at WISC-IV assessment in the CAP Dresden electronic health records. Although, we assume that most of the children receive medication only after the entire diagnostic procedure is completed at CAP, it cannot be ruled out that some children were under medication at WISC-IV assessment. This is important to note, because it is well known that stimulants could significantly influence the WISC performance and other cognitive measures [63, 64]. Here it must be considered, that the ADHD group had an FSIQ = 99 which was significantly above the FSIQ reported in meta-analytic studies of individuals with ADHD (e.g. [40]). Moreover, Khalifa et al. reported that medication in TS may result in lower WISC performance [20]. Thus, systematic examination of any medication is recommended for further studies and our results need to be interpreted in light of this limitation.

In our clinical data that were derived from the CAP Dresden electronic health records, no systematic information was available regarding symptom severity, age of onset, socio-economic status, and migration background. Thus, it was not possible to control for these factors. However, most children consulting the CAP Dresden live in Saxony (Germany) and the migration background can be assumed to be at a low level (Saxony statistics quantified the migration background in pupils at 9.7% in the year 2017 [65]).

The WISC-IV allows to analyze the intellectual profile of children in more detail, for example the General Ability Index (GAI: comprises the verbal comprehension and perceptual reasoning subtests and reflects reasoning abilities), the Cognitive Proficiency Index (CPI: includes the working memory and processing-speed subtests to evaluate proficiency and efficiency of cognitive processing) as well as FSIQ- GAI discrepancy and GAI-CPI discrepancy. However, these scores are not provided within the clinical routine and standard WISC-IV assessment at CAP and the focus of the present study was on the clinical routine and the main indices and FSIQ.

However, since the consideration of these scores in clinical care can give relevant information about intellectual functioning of developing children and children with neuro-developmental disorders such as the examined psychiatric disorders CTD, OCD and ADHD (e.g. [66–69]), these scores should be systematically considered in further studies as argued by different authors [67–69]. Especially the FSIQ should be interpreted with caution due to the influence of various factors, e.g. attention or executive deficits.

Finally, varying group sizes were predetermined by the clinical sample and the group OCD+ADHD (n = 4) had to be excluded from the statistical analyses for reason of statistical power. So it was not possible to apply a 3 × 2 factorial approach with the factors CTD (yes/no), OCD (yes/no) and ADHD (yes/no) that would allow drawing conclusions on the relevance of the non-comorbid and comorbid presentations of the considered disorders on intellectual ability. Although the size of the considered subgroups was sufficient in this study, analyses should be replicated within larger samples to verify our results. In addition, future studies could investigate the predictive or structural validity of the WISC-IV in more detail [59].

We excluded other comorbidities (e.g. adjustment disorder, anxiety disorder, mood disorder, autism spectrum disorder) from this sample to disentangle the specifics of intellectual ability in the mentioned disorders. However, future studies need to examine the role of further comorbidities, accordingly.

### Conclusion

Overall, we were able to confirm studies from the US on the intellectual ability in children with ADHD in a German sample. In addition, this study contributes new evidence on intellectual ability in children with non-comorbid and comorbid CTD and/or OCD from a German clinical sample. Children with CTD-only and OCD-only showed strengths in verbal comprehension. In clinical care and future research, comorbidity and medication needs to be considered and professionals have to be aware of strengths and deficits in WISC performance in children who are affected by the examined disorders since intellectual ability is an important factor for academic development, social functioning and well-being. Thus, the WISC-IV profile can be an important piece of the puzzle within a comprehensive psychological assessment that includes also a clinical interview, rating scales, observations, and cognitive measures.

## Data Availability

The datasets during and/or analysed during the current study available from the first author Sina Wanderer on reasonable request.
